# Validity and Reliability of the Turkish St. Mark’s Incontinence Score

**DOI:** 10.5152/tjg.2026.25551

**Published:** 2026-02-19

**Authors:** Özdal Ersoy, Yasemin Ecem Temel

**Affiliations:** 1 Division of Pelvic Floor Diseases and Bowel Health, Department of Gastroenterology, Arel University Memorial Health Group-Memorial Bahçelievler Hospital, İstanbul, Türkiye; 2 Department of Occupational Therapy, İstanbul Atlas University Faculty of Health Sciences, İstanbul, Türkiye

**Keywords:** Fecal incontinence, Incontinence Score, reliability, St. Mark’s (Vaizey), Turkish, validity

## Abstract

**Background/Aims:**

The St. Mark’s Incontinence Score (SMIS) is a widely used tool for assessing anal incontinence severity. This study aimed to evaluate the validity and reliability of the Turkish version of SMIS.

**Materials and Methods:**

A cross-sectional validation study with a prospective test–retest component was conducted among 60 patients with anal incontinence at 2 tertiary hospitals in Istanbul, Türkiye. The original SMIS was translated into Turkish and back-translated according to international translation guidelines. Content and construct validity were assessed by experts’ review and factor analysis. Reliability was evaluated using internal consistency (Cronbach’s alpha) and test–retest reliability.

**Results:**

As a result of the evaluations made in line with the opinions of the experts in the “continence field,” it has been shown that Turkish SMIS (T-SMIS) serves to measure anal incontinence with its content questions and is stated as valid. Reliability analyses indicated adequate internal consistency (Cronbach’s *α* = 0.76) and high test–retest reliability (intraclass correlation coefficient; *r= * 0.72-0.87, *P* = .01-.65).

**Conclusion:**

The T-SMIS is a psychometrically sound instrument for assessing fecal incontinence severity. Its adaptation into Turkish facilitates standardized clinical evaluation and international comparability. Notably, during the work, an Italian validation was published, highlighting the global applicability of SMIS across different cultures (Clinical trial registration: NCT05755763).

**Main Points**

- The Turkish version of the St. Mark’s Incontinence Score (T-SMIS) was developed and validated for patients with anal incontinence.
- The SMIS is a simple and widely used tool for evaluating incontinence, and its Turkish validation is crucial to ensure accessibility and use in daily clinical practice.
- This study confirms that T-SMIS is a valid and reliable tool, enabling standardized evaluation and international comparability in clinical practice.

## Introduction

While fecal incontinence (FI) is the involuntary leakage of mucus, liquid, or solid stool, anal incontinence refers to the involuntary leakage of gas in addition to these conditions.^1^

It has been shown that the frequency of FI among individuals aged 55-74 years in Türkiye is 5.2% and 13.2% in older age groups.^2^ This indicates that FI is a significant health problem in the country, and accurate measurement with validated tools is essential for proper assessment. Many scoring systems are used in the evaluation of anal incontinence.^3^^-^^5^ When it comes to scoring systems, the scales are reported as representative of the basic technique for the assessment of anal incontinence. The most commonly used scales in the clinic are the Wexner score, the FI Quality of Life Scale (FIQL), SMIS, and the FI Severity Index (FISI) for incontinent patients. The apparent heterogeneity of studies involving different scientific fields, evaluating different patient populations, and using different assessments complicates the methodology of studies investigating the applicability of anal incontinence scales. On the other hand, the fact that the scales are based on the subjective responses of the patients can also lead to evaluation bias. To overcome these limitations, the psychometric properties of the scales such as reliability, validity, sensitivity, and responsiveness are evaluated. The St. Mark’s (Vaizey) Incontinence Score (SMIS) is the one that most closely reflects the clinical findings of the physician regarding the degree of anal incontinence in those who are referred for anorectal physiologic examination and who complain of anal incontinence. The validated SMIS^6^ is a generic tool that can be used in different scientific fields and different populations and focuses on the type (solid, liquid, or gas) and frequency of anal incontinence, the influence of daily life, the need to use pads or plugs, the need for constipating medication, and the presence of urgency. To contribute to the creation of a common medical language in the local use of scales developed in different languages and cultures in medicine, scale adaptations are made through validity and reliability studies.^7^

SMIS is one of the scales used as a comparison tool in studies where other incontinence scales are translated into different languages.^8^ This scale stands out because it can be completed quickly and consists of 7 Likert-type questions divided into 2 sections. Questions about the disease’s status are asked in the first section, while questions concerning potential lifestyle modifications brought on by the illness are asked in the second.^6^ Its application period is short, and it is patient and practitioner friendly in clinical use.^9^ Although SMIS is validated in English and other populations, no Turkish version previously existed. This study aimed to perform the validity and reliability analysis of the Turkish version of the SIMS (T-SMIS) in patients with anal incontinence as well as to allow researchers to collect and compare Turkish data in a worldwide in a worldwide and intercultural context. Notably, during the work, an Italian validation was published highlighting the global applicability of SMIS across different cultures.^10^

## Materials and Methods

### Overall Study

This study was conducted as a cross-sectional validation study with a prospective retest design and was approved by the ethics committee of University of Acıbadem University (approval number 2016-15/7, date September 22, 2016). Written informed consent was obtained from patients who accept to enter into the study. The STROBE checklist for cross-sectional studies was used when reporting the results in the report.^11^

### Sample and Setting

This prospective cross-sectional validation study included 72 consecutive patients referred for anal incontinence to Fulya Acibadem Hospital and Acıbadem University Atakent Hospital. After exclusions, attrition, and the acquisition of signed informed the signed informed consent, 60 participants completed the study. Inclusion criteria were age ≥18 years and ability to understand Turkish. Patients with severe cognitive impairment or language difficulties were excluded.

**Figure 1.**
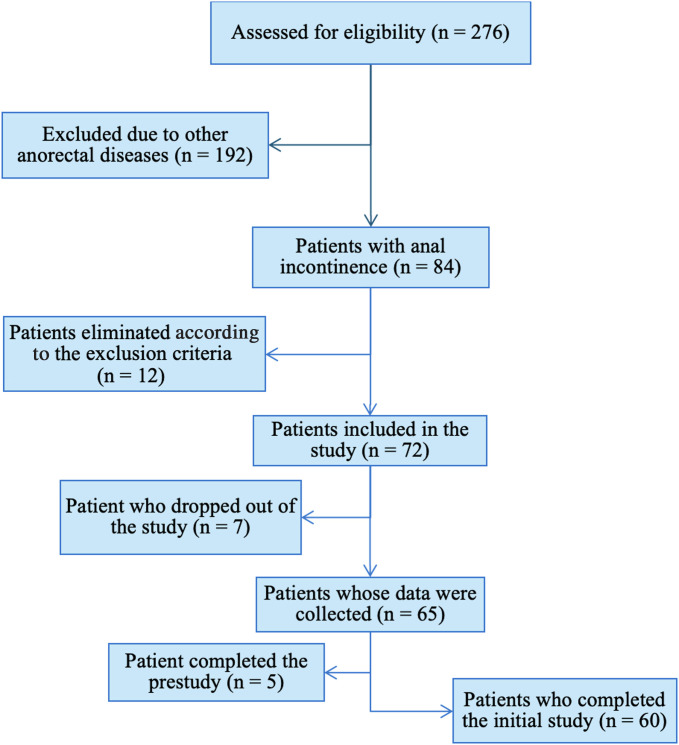
The flow chart of the study.

According to Gözüm and Aksayan, the recommended sample size in scale adaptation studies should be 5-10 times the number of items in the scale.^12^ Based on this guideline, the required sample size for the study was calculated to be between 35 and 70 participants. Ultimately, a total of 72 participants were prospectively enrolled, all of whom initially completed face-to-face consultations. During follow-up, 7 participants were lost, and 5 out of the remaining 65 were excluded from the analysis as they had been part of the pre-test group. The final analysis therefore included 60 participants. The flow chart of the study is presented in Figure 1.

### Instruments

#### Description of the Scale:

The SMIS has 2 parts. The first part includes 4 Likert-type questions on solid stool incontinence, liquid stool incontinence, gas incontinence, and lifestyle changes. Each item is scored from 0 to 4 with the following options: Never (no episodes in the past 4 weeks), Rarely (1 episode in the past 4 weeks), Sometimes (more than 1 episode in the past 4 weeks but less than once per week), Weekly (1 or more episodes per week but less than once daily), and Daily (1 or more episodes per day).

The second part evaluates 3 items: the use of pads or plugs, the use of constipating medication, and the ability to delay defecation for 15 minutes. These are scored as 0-2, 0-2, and 0-4, respectively.

The total score ranges from 0 to 24, with 0 indicating perfect continence and 24 indicating complete incontinence. Higher scores represent greater severity of incontinence. The Turkish version was adapted from the original SMIS.^6^

#### The Translation Process of the Scale:

The translation followed international guidelines.^13^ The scale was first translated into Turkish independently by 2 researchers whose native language was Turkish. A single consensus version was then created. This version was back-translated into English by 2 bilingual experts, and a common back-translation was produced. The back-translated version was compared with the original scale, and differences in meaning were reviewed. Based on this process, the Turkish version of the SMIS (T-SMIS) was finalized.

Before the main study, the T-SMIS was tested on 5 individuals to assess clarity and comprehensibility. Minor adjustments were made according to their feedback. Data from these pilot participants was not included in the final analysis.

#### Study Procedures:

The first interview was conducted face-to-face at the outpatient clinic. A second interview was carried out 2 weeks later, either by telephone or email. The 2-week interval was chosen to be long enough for patients not to remember their previous answers, yet short enough to minimize changes in symptoms. Although the optimal interval between test and retest may vary depending on the construct, its stability, and the study population, a 2-week period is generally recommended.^14^ Each questionnaire was coded with a pseudo number, and all personal information was removed.

#### Outcome Measures:

The main outcome measure is to obtain Turkish validation and reliability of SMIS.

### Statistical Analysis

For content and construct validity, the opinions of 2 gastroenterologists and 1 general surgeon with expertise in anal incontinence were obtained. To assess the suitability of the data for factor analysis, the Kaiser–Meyer–Olkin (KMO) coefficient and Bartlett’s test of sphericity were calculated. These results were then used to perform factor analysis.

Reliability was evaluated using Cronbach’s *α* coefficient. A value ≥0.9 was considered excellent, ≥0.8 good, and ≥0.7 acceptable.^15^

Test–retest reliability was examined by comparing the mean scores of the first and second administrations using paired *t*-tests. All statistical analyses were performed with SPSS Statistics for Windows, version 26.0 (IBM SPSS Corp.; Armonk, NY, USA).

## Results

Excluding the pre-test group, 36 men and 24 women completed the study (Table 1). The KMO coefficient was close to 1, and Bartlett’s test of sphericity was significant (*P* < .05) (Table 2).

Exploratory factor analysis revealed a 2-factor structure; Factor 1 represented symptom severity (solid, liquid, gas incontinence and pad use), Factor 2 represented behavioral/lifestyle impact (urgency, lifestyle changes, and constipating medication use). Factor analysis showed that 2 factors had eigenvalues greater than 1, with all 7 items loading onto these 2 factors (Table 3). The first factor explained 52.33% of the total variance, and the second factor explained 17.17%, accounting for 69.5% together (Table 4). The constipating medication item showed a negative loading (−0.67), consistent with its inverse relationship to severity.

Items related to liquid stool incontinence, solid stool incontinence, the use of pads/plugs, and gas incontinence loaded onto the first factor. Constipating medication showed a negative loading, suggesting this item behaves inversely in relation to symptom severity, yet conceptually aligns with the second factor.

### Reliability Analysis

Cronbach’s *α* was 0.87 for Factor 1 and 0.72 for Factor 2. The total scale had a coefficient of 0.76, indicating good reliability (Table 5).

### Test–Retest Comparison

Paired *t*-test analysis showed significant differences between the first and second measurements for solid stool incontinence, liquid stool incontinence, and lifestyle changes. In all 3 items, the mean score of the first test was higher than that of the retest (Table 6). Intraclass correlation coefficients (ICCs) ranged from 0.72 to 0.87 across SMIS items, indicating good overall test–retest reliability. Detailed ICC values are presented in Table 7. The ICC values reflect test–retest reliability.

## Discussion

Anal incontinence is defined as the involuntary loss of liquid or solid stool and/or gas.^1^^,^^16^ Several scoring systems are used to assess its severity. Bols et al^17^ evaluated the responsiveness of SMIS, the Wexner score, and the FIQL in a randomized clinical trial. Although none achieved high psychometric standards, all demonstrated acceptable external responsiveness and interpretability. Johannessen et al^18^ later reported that SMIS can be applied both as an interview-based and a self-administered tool, which increases its practicality in clinical use.

Anal incontinence is common and disabling, and may occur in patients with inflammatory or non-inflammatory bowel disease, as well as in those with obstetric anal sphincter injury. D’Amico et al^19^ investigated FI in patients with IBD and recommended the use of Wexner score as the first-line instrument.^19^ While the Wexner score focuses mainly on sphincter-related symptoms, SMIS adds items on urgency and constipating drug use, making it more comprehensive.^20^

Fecal incontinence may also result from other benign and malignant anorectal diseases. Despite its impact, the condition is often underdiagnosed due to patient reluctance to report symptoms and lack of standardized evaluation by clinicians. The Pelvic Floor Disorders Consortium recently recommended using both the Wexner and SMIS scores in practice, but there are no data on patient preferences when completing multiple questionnaires at the same time.^21^

In Türkiye, SMIS had not previously been translated or validated. The Wexner score was adapted into Turkish by Cam et al,^22^ but only in women. The FISI was also validated and found reliable in the Turkish population.^23^ For this reason, the SMIS was adapted into Turkish, and its psychometric properties were assessed.

The study showed that SMIS is suitable for evaluating the severity of anal incontinence and treatment outcomes. Recently, an Italian group also validated SMIS, further supporting its international applicability.^10^ It is believed that T-SMIS will improve the assessment of patients across specialties involved in anorectal disorders.

The total SMIS score ranges from 0 (full continence) to 24 (complete incontinence). Previous reports classified severity into 3 categories: 0-4, 5-8, and >8. In this study, the cross-cultural adaptation followed the standard translation–back translation method to ensure semantic and conceptual equivalence.^24^ Administration was performed both face-to-face and as as a self-report, which was considered superior to telephone application. Sixty-five patients met inclusion criteria; 5 were used for pre-testing, and 60 were included in the final analysis. The validated T-SMIS is presented in the Appendix.

Reliability refers to the extent to which an instrument measures consistently. Internal consistency is considered good when Cronbach’s *α* is >0.70.^25^ In the study, Cronbach’s *α* was 0.76, confirming reliability. Test–retest reproducibility was also high (*r* = 0.72-0.87, *P* = .01-.65). Validity was confirmed through expert review and factor analysis. Although overall test–retest reliability was good based on ICC, paired *t*-tests revealed small but statistically significant differences in solid stool, liquid stool, and lifestyle items shown in Table 6. This may reflect natural day-to-day fluctuations in FI symptoms, which are known to vary with diet, bowel habits, and psychological state. Therefore, the observed changes may be attributable to clinical variability rather than measurement instability.

This study has some limitations. Although both participating hospitals are located in the same city and belong to the same healthcare group, the sample was still derived from 2 tertiary referral centers, which may introduce referral or selection bias and limit the generalizability of the findings to broader community populations. In addition, although ICC values demonstrated good test–retest reliability, significant differences observed in several item-level scores between the test and retest suggest that the absolute stability of the scale may be influenced by natural day-to-day symptom fluctuations or potential recall effects. Another methodological limitation is that sensitivity to change was not assessed; therefore, it remains uncertain whether the T-SMIS can effectively detect clinical changes over time, although this property has been demonstrated in other validation studies. Furthermore, the study did not include convergent validity testing against other established incontinence instruments (e.g., the Wexner score, FIQL) within the same cohort. Future studies should incorporate comparison scales and evaluate responsiveness in longitudinal clinical settings to strengthen the psychometric profile of the T-SMIS.

In conclusion, this study demonstrated that the Turkish version of the SMIS is valid and reliable. The Cronbach’s *α* value of 0.76 confirms internal consistency, and test–retest results indicate high reproducibility. T-SMIS can be used for the evaluation and follow-up of anal incontinence in Turkish-speaking patients.

## Figures and Tables

**Table:** 

	Asla	Nadiren	Sık sık	Haftalık	Günlük
Katı dışkı tutamama	0	1	2	3	4
Sıvı dışkı tutamama	0	1	2	3	4
Gaz tutamama	0	1	2	3	4
Hayat tarzında değişiklik yapma ihtiyacı	0	1	2	3	4
				Hayır	Evet
Ped/tıkaç kullanma ihtiyacı				0	2
Kabız yapıcı ilaç kullanma ihtiyacı				0	2
Dışkıyı 15 dakika erteleyememe				0	4

**Table 1.** Characteristics of the Patients

	n (%)	Age (Years) Mean (SD)
Women	24 (40%)	48.66 (9.27)
Men	36 (60%)	49.36 (8.7)
Total	60	49.08 (1.14)

SD, standart deviation.

**Table 2.** KMO Coefficient and Bartlett Sphericity Test

KMO		0.71
Bartlett sphericity test	Chi-square	229.15
	SD	21
	*P*	.000

KMO, Kaiser-Meyer-Olkin; SD, standart deviation.

**Table 3.** Factor Analysis

Factor	Initial Eigenvalues			Sum of Squares		
	Total	Variance %	Cumulative %	Total	Variance %	Cumulative %
1	3.66	52.33	52.33	3.66	52.33	52.33
2	1.2	17.17	69.5	1.2	17.17	69.5
3	.94	13.48	82.98			
4	.47	6.74	89.73			
5	.41	5.92	95.66			
6	.2	2.92	98.58			
7	.09	1.41	100			

**Table 4.** Factor Weight Matrix

	Factor	
	1	2
Liquid stool incontinence	0.93	
Solid stool incontinence	0.89	
Need to use pad/plug	0.75	
Gas incontinence	0.74	
Need to make changes in lifestyle		0.75
Not being able to delay stool for 15 minutes		0.69
Need to use constipating medication		−0.67

**Table 5.** Cronbach Alpha Coefficient

	Cronbach *α* Coefficient
Total	0.76
Factor 1	0.87
Factor 2	0.72

**Table 6.** Dependent Groups *t*-test

		n	Mean	SD	*t*	*P*
Solid stool incontinence	Test	60	1.27	1.35	2.56	.013*s
	Retest	60	1.17	1.30		
Liquid stool incontinence	Test	60	1.75	1.40	2.79	.007*
	Retest	60	1.63	1.46		
Gas incontinence	Test	60	2.13	1.46	1.98	.05
	Retest	60	2.02	1.26		
Need to make changes in lifestyle	Test	60	2.67	1.45	2.05	.04*
	Retest	60	2.47	1.42		
Need to use pad/plug	Test	60	1.07	1.01	−1	.32
	Retest	60	1.13	1		
Need to use constipating medication	Test	60	1.62	0.72	0.44	.65
	Retest	60	1.58	0.74		
Not being able to delay stool for 15 minutes	Test	60	2.03	1.93	1.84	.07
	Retest	60	1.57	1.88		

**Table 7.** Intraclass Correlation Coefficients (ICC) Table for T‑SMIS

SMIS Item	ICC Value	95% CI	Reliability Interpretation
Solid stool incontinence	0.72	0.60-0.82	Good
Liquid stool incontinence	0.81	0.70-0.89	Good–Excellent
Gas incontinence	0.75	0.63-0.84	Good
Lifestyle changes	0.74	0.61-0.83	Good
Pad/plug use	0.70	0.55-0.80	Acceptable–Good
Constipating medication	0.72	0.59-0.82	Good
Ability to defer defecation for 15 minutes	0.87	0.79-0.93	Excellent

## Data Availability

The data that support the findings of this study are available on request from the corresponding author.

## Appendix

Turkish Version of The St.Marks’s Incontinence Questionnaire (The original English version can be found in reference 6).

St. Mark’s İnkontinans Skoru

İsim:

Tarih:

Asla: Son 4 hafta içinde hiç olay yok.

Nadir: Son 4 hafta içinde 1 olay var.

Sık sık: Son 4 hafta içinde 1den fazla ancak haftada 1den az olay var.

Haftalık: Haftada 1 veya daha fazla olay var ancak günde 1den az.

Günlük: Günde 1 veya daha fazla olay var.

Minimum skor: 0 mükemmel kontinans

Maksimum skor: 24 total inkontinans
